# Specific gFET‐Based Aptasensors for Monitoring of Microbiome Quality: Quantification of the Enteric Health‐Relevant Bacterium *Roseburia Intestinalis*

**DOI:** 10.1002/adhm.202403827

**Published:** 2024-12-11

**Authors:** Yiting Zhang, Hu Xing, Runliu Li, Jakob Andersson, Anil Bozdogan, Robert Strassl, Bastian Draphoen, Mika Lindén, Marius Henkel, Uwe Knippschild, Roger Hasler, Christoph Kleber, Wolfgang Knoll, Ann‐Kathrin Kissmann, Frank Rosenau

**Affiliations:** ^1^ Institute of Pharmaceutical Biotechnology Ulm University Albert‐Einstein‐Allee 11 89081 Ulm Germany; ^2^ AIT Austrian Institute of Technology GmbH Giefinggasse 4 Vienna 1210 Austria; ^3^ Division of Clinical Virology Medical University of Vienna – Spitalgasse 23 Vienna 1090 Austria; ^4^ Institute of Inorganic Chemistry II Ulm University Albert‐Einstein‐Allee 11 89081 Ulm Germany; ^5^ Cellular Agriculture TUM School of Life Sciences Technical University of Munich Gregor‐Mendel‐Str. 4 85354 Freising Germany; ^6^ Department of General and Visceral Surgery Surgery Center Ulm University Albert‐Einstein‐Allee 23 89081 Ulm Germany; ^7^ Danube Private University Steiner Landstraße 124 Krems an der Donau 3500 Austria

**Keywords:** aptamer, rGO‐FET, roseburia intestinalis, SELEX

## Abstract

*Roseburia intestinalis*, enriched in the gut, is closely associated with obesity, intestinal inflammation, and other diseases. A novel detection method for *R. intestinalis* to replace the commonly used 16S rRNA sequencing technique is aim to developed, thus enabling real‐time and low‐cost monitoring of gut microbiota. The optimal solution is to utilize rGO‐FET (reduced graphene oxide field‐effect transistor) functionalized with aptamers. Due to the high sensitivity of graphene sensors to electronic changes in the system, it is anticipated to achieve detection sensitivity that traditional fluorescence detection techniques cannot attain. The previous work reported a nucleic acid aptamer library, Ri 7_2, capable of quantitatively tracking *R. intestinalis* in complex systems. However, due to the complexity of the aptamer library itself, large‐scale industrial synthesis is challenging, significantly limiting its further commercial application potential. Therefore, in this study, through Next‐Generation Sequencing analysis, four representative single aptamers from the aptamer library is strategically selected, named A‐Rose 1, A‐Rose 2, A‐Rose 3, and A‐Rose 4, and confirmed their excellent performance similar to the aptamer library Ri 7_2. Furthermore, aptamer‐modified rGO‐FET demonstrated universality in detecting *R. intestinalis* in a series of biochemical analyses, providing a novel and powerful diagnostic tool for the clinical diagnosis of *R. intestinalis*.

## Graphical abstract

Aptamer‐modified rGO‐FET biosensing demonstrated universality in detecting *Roseburia intestinalis*. The aptamers allowed specific quantification and discrimination of the desired target cells from other bacterial control cells with a lower detection limit of 10 cells mL^−1^. Even in in the background of human stool samples, alterations of in the abundance of *R. intestinalis* in the range of 1% could easily be retraced.

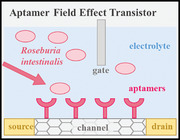

## 1 Introduction

*Roseburia intestinalis*, an anaerobic, Gram‐positive, butyrate‐producing bacterium, has been found to be beneficial in maintaining intestinal health and treating diseases. It produces the essential short‐chain fatty acid butyrate, which plays a central role in cell differentiation and apoptosis, promoting the differentiation of Treg cells and IL‐10‐producing T cells, which improves intestinal inflammation. *R. intestinalis* has been shown to suppress inflammation in ulcerative colitis and inhibit the development of atherosclerosis in mouse models.^[^ ^1^ ^]^ Studies have shown that this bacterium can ameliorate colitis, alcohol‐related liver diseases, and nonalcoholic fatty liver disease. It has also been found to alleviate symptoms of type 2 diabetes by increasing the production of short‐chain fatty acids (SCFAs). *R. intestinalis* abundance has also been found to be lower in the gut of colorectal cancer patients, making it a reliable biomarker in the treatment of these conditions. Imbalances in the abundance of individual bacteria in the human gut (i.e., a significant increase or decrease causing or accompanying health threats), the “dysbiosis” within a given microbiome can serve as indication for health risks or even as a marker for the onset or progression of a (microbiome‐related) disease. The next “big thing” in preventive nutrition science and human medicine is the correction of dysbiosis by supplementing “therapeutic probiotics” (i.e., selected next‐generation bacterial strains with scientifically proven impact on the respective disease) or – more radical, but also more inconvenient for the patient – complete exchange of the gut microbiome by comprehensive antibiotic treatment and subsequent transplantation of “healthy” microbiomes via application of stool samples formulated in capsules to be ingested by the patients.^[^ ^1^, ^2^, ^3^, ^4^ ^]^ To assure the success of a probiotic supplementation therapy or to evaluate the efficiency of severe changes in the dietary regime on the development of a patient's microbiome toward a healthier status ideally requires a fast, reliable, easy‐to‐handle, and affordable monitoring technique. However, traditional bacterial detection methods such quantitative polymerase chain reaction (qPCR) and next‐generation sequencing (NGS) using primers to amplify 16S rRNA genes not only require complex equipment but are also time‐consuming and economically inefficient.^[^ ^5^, ^6^, ^7^ ^]^ NGS especially – depending on the particular service provider and the conditions and the service contract – can have processing times in the range of several weeks, which can practically contradict the principle advantage of the method to acquire extremely detailed quantitative information on the microbiome composition. This achievable broadness of quantitative data is simply not necessary in the case of known dysbiosis and in turn consequently not beneficial for monitoring, e.g., the success of probiotic supplementation therapies. Aptamers, i.e., single‐stranded oligonucleotides (DNA or RNA) as an alternative type of binding molecules, can specifically label whole living cells by recognizing distinct cell surface structures, thus being capable to detect and enable quantification of the dedicated target bacteria in complex systems including not only defined mixtures, but also human stool samples.^[^ ^8^, ^9^, ^10^ ^]^

Biosensing has already made use of the idea of electrolyte‐gated field‐effect transistors (EG‐FETs) in electrical devices. This method has been reported to enable the identification of microRNA,^[^ ^11^ ^]^ DNA as a biomarker in some cardiac disorders,^[^ ^12^, ^13^ ^]^ and tiny molecules such as urea or biotin.^[^ ^14^, ^15^ ^]^ Additionally, we have developed very sensitive approaches for the detection of retinol binding protein 4 as a possible new marker for the beginning of type‐2 diabetes^[^ ^16^ ^]^ and the E7‐protein for the human papillomavirus involved in carcinogenesis.^[^ ^17^ ^]^ Moreover, we have demonstrated that by an approach of the same type the gut bacterium *Blautia producta* could be quantified in stool samples with aptamer‐based FETs.^[^ ^18^ ^]^ The main benefit of EG‐FET‐based sensing devices is their high sensitivity, which can be achieved without the requirement for fluorescent or redox labels to modify the target.

**Scheme 1:**
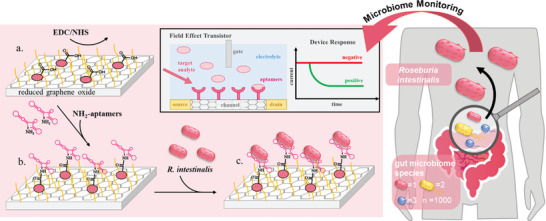
Human gut microbiome dysbiosis detection by aptamer‐based biosensing. Functionalization of rGO‐FET with anti‐Roseburia individual aptamers. a.) Chips were immersed in PyPEG (PEG pre‐conjugated with a PBSE) (500 mm) and 1‐pyrenecarboxylic acid (PCA, 50 mm, linker) to obtain a 9:1 ratio of blocking and linking agents on the reduced graphene surface. b.) 5′‐NH2‐modified aptamers (A‐Rose 1, A‐Rose 2, A‐Rose 3, and A‐Rose 4) immobilization by first activating the carboxyl groups using EDC (15 mm)/NHS (15 mm) for 30 min, followed by covalent coupling of the 5′‐NH2‐modified aptamers (100 nm in milliQ grade water for 1 h at 25 °C). c.) Specific affinity recognition of R. intestinalis cells. Inlay: Typical configuration of an rGO‐FET with response of the device when there is no target applied (red) and when the target analyte is present (green).

The sensor chips themselves and the (electronic) read‐out equipment are both inexpensive and widely accessible, which makes them perfect for future development to point‐of‐care settings, even in technologically less developed areas of the world, where access to laboratory conditions and expertise may be limited. The generic EG‐FET operates on the same concept as metal oxide FETs with a semiconducting channel dividing the source and drain electrodes (**Scheme** **1**). By applying a certain voltage at the gate electrode, changes of channel conductivity are induced thereby modulating the current between the source and drain electrodes. In principle, any binding molecule (e.g., protein‐ligands, antigens, traditional or alternative scaffold proteins including antibodies or 3D structured oligonucleotide aptamers) can represent functionalities of the modified channel. Driven by molecular interaction of the respective analyte molecules with the binding entity, the dielectric layers are changed in their structure thereby changing the distribution of charges at the channel and electrolyte interface, which then alters the propagation of the gate voltage, resulting in a change in charge carrier mobility.^[^ ^19^ ^]^ Binding events on the channel can be observed as changes in the source–drain current (I_DS_), when the gate voltage is kept constant. As we have described previously, already the focused aptamer libraries immobilized on reduced graphene oxide field‐effect transistors (rGO‐FETs, a sub‐class of EG‐FET devices)^[^ ^20^ ^]^ allowed discrimination of empty RBP4 from the loaded form (apo‐ vs holo‐RBP4) without the need of isolating individual aptamers prior construction of the chip.^[^ ^16^ ^]^ Here, *R. intestinalis*‐specific individual aptamers were isolated by bioinformatic analyses from the previously described final library Ri7_2^[^ ^8^ ^]^ after evaluating the functional sequence space of the library by NGS as described before for antibacterial aptamer isolation.^[^ ^21^, ^22^ ^]^ These four aptamers were initially characterized biochemically determining their affinities and proving their specificity to whole cells of the dedicated target species. They were used to functionalize rGO‐FETs to demonstrate that this concept of aptamer‐based electronic sensors is also suitable to quantify bacterial cells. We further show that *R. intestinalis* can be distinguished from control microbiome bacteria in synthetic bacterial mixtures with sufficiently high sensitivity and specificity. Moreover, it was possible to quantify *R. intestinalis* even in real human stool samples with its considerably high background of “contaminating” gut bacteria.

## 2 Results and Discussion

**Figure 1:**
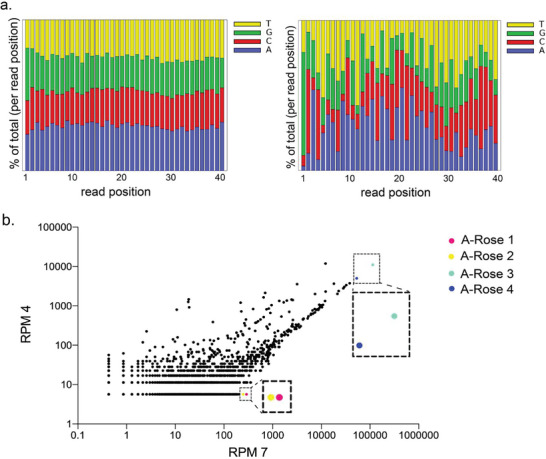
a). Nucleotide distribution in aptamer libraries Ri 4 and Ri 7_2 (from left to right). The distribution of each nucleobase is presented as a percentage: red‐cytosine (C), yellow‐thymine (T), blue‐adenine (A), and green‐guanine (G). b). Scatter plot of sequence responses in Ri 4 and Ri 7_2, with the progress of SELEX. The highest enriched single aptamers A‐Rose 1 and A‐Rose 2, and the two highest abundant single aptamers A‐Rose 3 and A‐Rose 4 were selected and are depicted in red for A‐Rose 1, yellow A‐Rose 2, green A‐Rose 3, and blue for A‐Rose 4.

**Figure 2:**
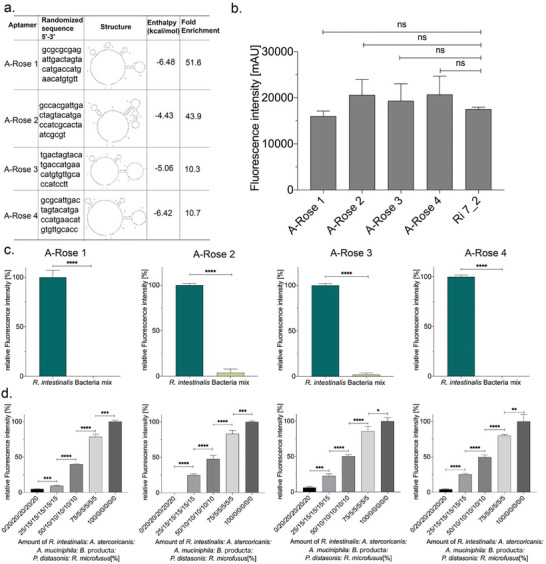
a). Random sequences, secondary structures, folding enthalpies, and enrichment rates relative to the Ri 4 aptamer library for single aptamers A‐Rose 1–4. b). Connectivity assay results targeting R. intestinalis show that single aptamers A‐Rose 1–4 exhibit binding abilities similar to those of the Ri 7_2 aptamer library, with no significant differences observed between groups. “ns” represents *p* < 0.05, indicating no significant difference. c). Specific detection: Cy5‐labeled aptamers were separately incubated with *R. intestinalis* and bacterial mix under the same conditions. d). Quantitative tracking of *R. intestinalis* in a complex microbial community: *R. intestinalis* was mixed with Bacteria mix at different ratios and co‐incubated with equal amounts of aptamers under the same conditions. All experiments were conducted in triplicate with error bars representing standard deviations. *p* values < 0.05 were considered significant. ^*^ *p* denotes < 0.05, ^**^ denotes *p *< 0.01, ^***^ denotes *p *< 0.001 and ^****^ denotes *p *< 0.0001.

Seven rounds of whole Cell‐SELEX against *R. intestinalis* as the target gut microbiome member delivered a focused (or polyclonal) DNA oligonucleotide aptamer library, which was sufficient to specifically label and discriminate *R. intestinalis* from other gut bacteria.^[^ ^8^ ^]^ Evaluating the sequence space of the final aptamer library Ri7_2 in comparison to the earlier round 4 by NGS was the foundation for the in‐depth analysis and subsequent isolation of individual aptamers. A commercial (and thus ideal) synthetic aptamer initial library must be expected to contain even distributions of all four nucleobases (adenosine, thymine, cytosine, and guanine) in the oligonucleotides’ sequences. Successive selection of sequences with increasing affinities toward the respective target leads to shifts within the distribution of individual nucleotides in the entire sequence space, which in turn can result in an increase of the GC‐content (IMPATIENT). This causes increasing aptamer melting temperatures, which can easily be measured by a novel quantitative PCR method, we have recently introduced and named “IMPATIENT‐qPCR”.^[^ ^23^ ^]^ The *R. intestinalis* round seven library (Ri7_2) showed a final melting temperature of 80 °C, which was determined previously (library and impatient). The Ri4 library showed a typical distribution of an early SELEX round, which was still close but identical to the ideal 25% each distribution (**Figure** **1**a, left), whereas in the final library Ri7_2, in contrast, the nucleotide composition significantly shifted as expected indicating a successful SELEX evolution and selection process developing higher affinity aptamers (Figure 1a, right). Plotting frequencies (reads per million (RPM)) of individual sequences occurring in the total sequence space of NGS sequencing reactions of early and final SELEX rounds delivers a graph showing individual sequence possessing same abundancies the compared samples on a diagonal line. This plot delivers sequences with both, the highest total abundancy in the final round and the most enriched aptamers between early and final rounds during the evolution process (Figure 1a). As the most enriched aptamer group the sequences of A‐Rose 1 and A‐Rose 2 arose from the library, whereas the highest abundance aptamers were represented by A‐Rose 3 and A‐Rose 4 (Figure 1b). These individual sequences were chemically synthesized to enable their further biochemical characterization as binding molecules, which also included predictions of most probable secondary structures with their theoretical stabilities (folding enthalpy) via the Mfold web server established for such predictions of oligonucleotide folding and intramolecular hybridization by Jerry Zuker more than twenty years ago^[^ ^24^ ^]^ (**Figure** **2**a).

These four individual aptamers were fluorescently labeled with Cyanine 5 (Cy5) as described for the binding studies of the *R. intestinalis* polyclonal aptamer library originating from the earlier described FluCell‐SELEX.^[^ ^8^ ^]^Using the library Ri7_2 as a reference all four aptamers kept the high affinity toward the dedicated target with only non‐significant differences to the library affinity (Figure 2b). In addition, they possessed highly significant specificities for *R. intestinalis*, when their cell‐labeling capabilities were compared using equal cell numbers of a designed “consortium” consisting of five mixed control bacteria (*Akkermansia muciniphila* (*A. muciniphila*), *Allobaculum stercoricanis* (*A. stercoricanis*), *Blautia producta* (*B. producta*), *Parabacteroides distasonis* (*P. distasonis*), and *Rikenella microfusus* (*R. microfusus*)) (Figure 2c), which also belong to the gut microbiome as prominent natural inhabitants and were used as controls previously.^[^ ^8^, ^9^, ^10^, ^21^, ^25^ ^]^ This was also the case in the experiments using the presence of this consortium as mixed “contaminating” bacterial cells in which the increasing cell numbers of *R. intestinalis* could be easily and reliably retraced (Figure 2d).

**Figure 3:**
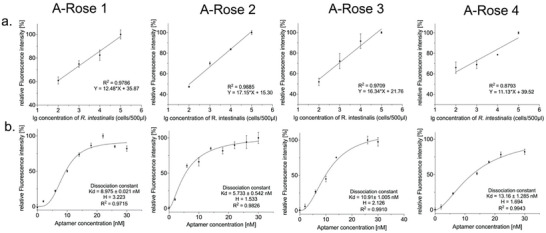
Characterization of single aptamers A‐Rose 1–4. Bacteria mix: A mixed microbial system of *A. muciniphila*, *B. producta*, *P. distasonis*, *A. stercoricanis*, and *R. microfusus* in equal proportions. *p*‐values < 0.05 were considered significant, where ^*^ represents *p* < 0.05, ^**^ represents *p* < 0.01, ^***^ *p* < 0.001, and ^****^ *p* < 0.0001. a). Sensitivity of detection: Equal amounts of aptamers were co‐incubated with different quantities of *R. intestinalis*, ranging from 10^2^ to 10^5^ bacteria per 500 µL. The change in fluorescence intensity was recorded and showed a significant positive linear correlation with bacterial quantity. The detection limits for A‐Rose 1–4 were determined to be 10^2^ (R^2^ = 0.9786), 10^2^ (R^2^ = 0.9885), 10^2^ (R^2^ = 0.9709), and 10^2^ (R^2^ = 0.8793) bacteria, respectively. b). Affinity detection: Different concentrations of aptamers were co‐incubated with equal amounts of *R. intestinalis*. Nonlinear regression curves were fitted using the specific binding with Hill slope model in GraphPad Prism 8, yielding dissociation constants (K_d_) for A‐Rose 1–4 of 8.975 nm (R^2^ = 0.9715), 5.733 nm (R^2^ = 0.9826), 10.91 nm (R^2^ = 0.9910), and 13.16 nm (R^2^ = 0.9943), respectively, with Hill coefficients (H) of 3.223, 1.533, 2.126, and 1.694. All experiments were conducted in triplicate with error bars representing standard deviations.

Detection limits were found to be between 200 cells mL^−1^ for A‐Rose 1–4, respectively (**Figure** **3**a). The fluorescence‐based preliminary performance characterization of the selected individual aptamers was finalized by determining the dissociation constants (K_d_). The K_d_‐values deduced from the binding experiments with increasing amounts of aptamers ranged from 5.7 to 13.2 nm (A‐Rose 2<A‐Rose 1<A‐Rose 3<A‐Rose 4) (Figure 3b) and hence are lying in the same range as the K_d_ value of the original polyclonal library (9.7 nm).^[^ ^8^ ^]^

**Figure 4:**
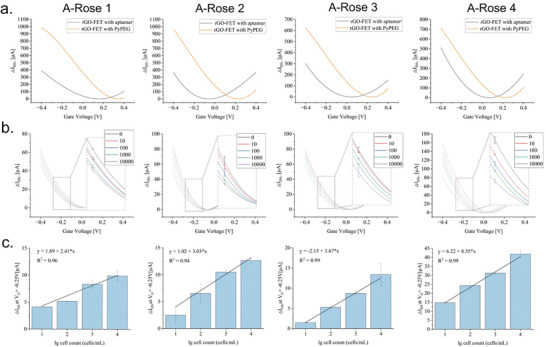
Determination of specificity and sensitivity of rGO‐FET modified by single aptamers A‐Rose1–4. a). Verification of aptamer functionalization of the rGO‐FET surface by comparison of shifts in the I_Ds_V_G_ curves before and after aptamer immobilization. b). The specific binding events of rGO‐FET modified by different aptamers to *R. intestinalis*. c). The relationship between ΔI_DS_ and the change in the number of bacteria in the analyte for different binding events when the gate voltage (VG) is −0.25 V. All experiments were conducted in triplicate with error bars representing standard deviations.

The aptamer functionality as specificity determining molecules on rGO‐FET was evaluated upon coupling with EDC‐NHS (Scheme 1) as described previously for proteins^[^ ^16^, ^26^ ^]^ and cells^[^ ^18^ ^]^ and implementing the established gate‐voltage (V_G_) scanning protocol from −0.5 to +0.5 V.^[^ ^27^ ^]^ The commercial chips were coated with graphene, subsequently chemically reduced by hydrazine treatment followed by coating with PyPEG (providing accessible carboxy‐groups) upon quality control using Raman spectroscopy (Figure S1, Supporting Information) and microscopy (Figure S2, Supporting Information). The sequential functionalization of each chip surface with amine‐labeled aptamers upon EDC/NHS activation (“layer‐by‐layer” functionalization) was characterized by cyclic voltammetry prior to the respective individual quantitative sensor measurements (**Figure** **4**a). The aptamer‐modified FETs were able to capture the dedicated target cells present in the samples as expected. This resulted in the induction of distinct shifts in the I_D_V_G_ curves by altering the charge distribution within the semiconductor structure. Recording I_Ds_V_G_ curves of samples containing increasing numbers of *R. intestinalis* cells (0–10 00000 cells mL^−1^) revealed increased shift alterations (ΔI_DS_) depending on the increase in cell numbers evaluated at constant V_G_ conditions (= −0.25 V), fitting distinctive linear progression functions for the aptamers A‐Rose 1–4 with reasonable determination coefficients (R^2^) (Figure 4b,c).

**Figure 5:**
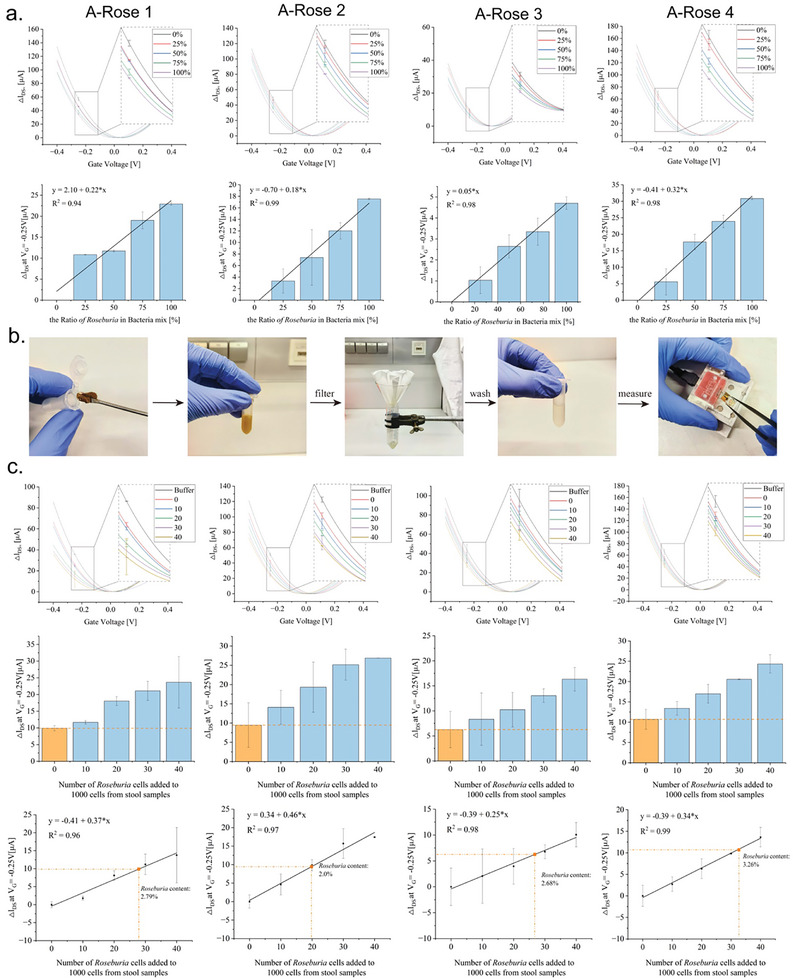
Quantitative Tracking of *R. intestinalis* using gFET Modified with single aptamer A‐Rose 1–4. a). Quantitative tracking of R*. intestinalis* in artificially constructed mixed‐bacteria contamination model. The top row of graphs details the binding events between rGO‐FETs modified with four different aptamers and varying proportions of *R. intestinalis* in the mixed bacteria model. The mixed bacteria model consists of *P. distasonis, A. muciniphila, B. producta, A. stercoricanis*, and *R. microfusus* distributed proportionally. With a constant presence of 100 bacteria per 1 mL, changes in I_D_V_G_ curves are recorded as *R. intestinalis* occupies 0%, 25%, 50%, 75%, and 100% of the mixed system. The bottom row graphs record the ΔI_DS_ shift distance between different models at VG of −0.25 V. b). The extraction of fecal bacteria involves dissolving human feces in 1× PBS buffer, filtering to remove insoluble solid impurities, and subsequently obtaining fecal bacteria through centrifugation and washing, which are then used as analytes for rGO‐FET biosensing. c). Quantitative tracking of *R. intestinalis* in fecal bacteria. The top row of graphs reports the binding events between rGO‐FETs modified with four different aptamers and varying quantities of *R. intestinalis* in the background of constant amounts of fecal bacteria. The total fecal bacteria count was fixed at 1000, and 0, 10, 20, 30, and 40 *R. intestinalis* were gradually added to build a complete incremental model of *R. intestinalis* in fecal bacteria. The bottom row graphs show the ΔI_DS_ shift between different models at VG of −0.25 V with positive linear correlation between the shift distance of I_D_V_G_ curves in different models and the increment of *R. intestinalis*. The y‐coordinate of the points marked on the regression line represents the ΔI_DS_ shift triggered by 1000 fecal bacteria, while the x‐coordinate represents the quantity of *R. intestinalis* in the sample. The detection results from gFETs modified with four aptamers A‐Rose 1–4 indicate the content of *Roseburia* in the volunteers’ feces to be 2.79%, 2.0%, 2.68%, and 3.26%, respectively. All experiments were conducted in triplicate with error bars representing standard deviations.

In these experiments samples were adjusted to calculated numbers of *R. intestinalis* cells and delivered unambiguous signals that were interpreted as the lower detection limits for the four aptamers (Figure 4b,c). Here, on the functionalized gFETs, the lower detection limit was found to be 10 cells mL^−1^ in total of *R. intestinalis*, whereas the limits in the fluorescence‐based assay were 200 cells/mL, suggesting a gain of sensitivity for the gFET‐aptasensors up to 20‐fold, respectively. The specificity of these gFETs was analyzed in defined mixtures consisting of a total of 100 bacterial cells, where *R. intestinalis* comprised either 0%, 25%, 50%, 75%, or 100% of all cells and the remaining mixture was composed of an otherwise constant number of *P. distasonis, A. muciniphila, R. microfusus, A. stercoricanis*, and *B. producta* cells (1:1:1:1:1). Again, an increase of the numbers of *R. intestinalis* cells present in the samples could be followed and according linear progression curves could be defined with R^2^‐values above 0.94, 0.99, 0.98, and 0.99 for the aptamers A‐Rose 1–4, respectively (**Figure** **5**a).

With the aim to demonstrate the functionality of the gFET‐based measurements of *R. intestinalis* in the background of contaminating bacteria also in the far more complex background of real stool samples the experimental set‐up was used with real samples of a healthy male volunteer. Therefore, bacteria were released to their free‐floating form (i.e., the “planktonic phase”) from the fecal samples, to get access to the microbiome, by removing clotted solid matter from the raw material upon dissolution in 1× PBS buffer (the work‐flow is illustrated in Figure 5b). Cell densities were determined spectrophotometrically by measuring the optical density at a wavelength of 600 nm (O.D._600_) and adjusted to a calculated number of 1000 of fecal bacterial cells per 1 mL sample. This constant number of cells was then enlarged by gradual addition (“spiking”) of *R. intestinalis* cells to the sample at calculated numbers of 0, 10, 20, 30, and 40. The specific increases correlated linearly with the shift increases of the ΔI_DS_ curves detected by the gFET‐aptasensors (Figure 5c). Using the respective individually derived linear progression functions the original content of *R. intestinalis* cells in the stool sample could be derived to be in the range between 2.0% and 3.3% (mean = 2.7%) for the four individual aptamers (Figure 5c). These values fit the amount of *R. intestinalis* known from literature to account for a known but at least considerable broad and thus uncertain range of 0.9%–5.0% (mean = 2.3%) in the human gut microbiome.^[^ ^28^, ^29^, ^30^ ^]^ We have analyzed stool samples of the respective volunteer twice^[^ ^8^ ^]^ (see Xing et al. 2022 and Table S1, Supporting Information) by NGS and found the content of 4.8% for *Roseburia* spec. constant over a period of more than two years. To explain this higher value the fact has to be considered that NGS sequencing as it was performed in the study does not analyze the sequence data down to the species level, but stops already at the genus level thereby not differentiating other *Roseburia* species. The gFET measurements based on aptamers derived particularly against *R. intestinalis* may open opportunities to differentiate the bacteria below the genus level upon characterization of the sensors for specificity and sensitivity against sub‐genus level bacteria, which may be an attractive task for a respective follow‐up study.

Since its initial discovery in 2002, *R. intestinalis* has garnered extensive research attention.^[^ ^31^ ^]^ *R. intestinalis* is one of the most common bacterium in the gut and is an important producer of short‐chain fatty acids (SCFAs), such as butyrate. Notably, butyrate has been demonstrated to prevent intestinal inflammation and helps to maintain energy homeostasis.^[^ ^32^, ^33^ ^]^ Substantial scientific evidence suggests that decreased abundance of *R. intestinalis* in the gut may lead to conditions such as inflammatory bowel disease (IBD) [including Crohn's disease (CD) and ulcerative colitis (UC)],^[^ ^8^, ^28^, ^34^, ^35^ ^]^ diabetes,^[^ ^36^, ^37^ ^]^ and colorectal cancer.^[^ ^38^, ^39^ ^]^ In recent years, strategies involving *R. intestinalis* have been applied as adjunctive therapies for related diseases, with fecal microbiota transplantation (FMT) being highlighted as particularly important.^[^ ^28^ ^]^ FMT, as a standard therapy, has shown significant positive effects in restoring a healthy gut microbiota and improving disease pathophysiology. However, the success of FMT and probably of future supplementation strategies with cultivated *R. intestinalis* as a next‐generation probiotic, depends on the restoration of *R. intestinalis* to a “healthy” and stable physiological abundance in the gut. This subtle adjustment of the gut microbiome must be expected to be a rather longsome process and to require easy but precise monitoring to prove its success. Therefore, based on two very well‐established technologies, the aptamer, and the gFET methodology, we have developed here a novel gFET‐based electronic aptasensor and proofed its functionality for specific monitoring of *R. intestinalis* in the gut microbiome.

Intending to develop a general monitoring technique to support directed therapeutic microbiome engineering approaches by supplementing selected individual strains or consortia of microorganisms the novel technique may be interpreted as an important milestone, since its performance meets the requirements for sensitivity and specificity to measure microbe abundance. *R. intestinalis* belongs to the 20 most abundant (and probably most relevant) genera in a healthy microbiome and thus represents a reasonable benchmark of bacterial quantities to be measured in the future also for other gut bacteria.^[^ ^28^ ^]^ In addition, gut microbiota quantification measurements have to be specific enough for the desired target microorganism, i.e., the sensor and in turn, the aptamer chosen as a binding entity has to “ignore” contaminating cells being simultaneously present in the (stool) sample to be analyzed. Electronic sensors based on rGO‐FETs have been presented against prominent pathogens including the SARS‐Cov‐2 virus and bacterial pathogens like *Stahphylococcus aureus* and *Acinetobacter baumanii* based on biofunctionalization with antibodies or designed peptides.^[^ ^40^, ^41^ ^]^ Moreover, biosensors for the detection of *Escherichia coli* have been described based on an individual pyrene‐modified aptamer,^[^ ^42^ ^]^ however, the aptamer used was at best mediocre (>25 nm)^[^ ^43^ ^]^ as compared to A‐Rose 1–4. Moreover, the detection limit of this sensor was one order of magnitude higher and the degree of specificity for the discrimination of *E. coli* from other (bacterial) cells remained open.^[^ ^32^ ^]^

The individual aptamers A‐Rose 1–4 isolated from the *R. intestinalis*‐specific polyclonal aptamer library^[^ ^8^ ^]^ and characterized in this study proved that they can serve as valuable binding molecules for the construction of aptamer‐based gFETs and allow specific detection of the target bacterium not only in the presence of selected species in mixtures, but also in the highly complex background of a real stool sample. Based on this result in‐depth evaluation of this novel concept appears to be promising involving validation of the sensor results with comparative NGS‐based quantification of *R. intestinalis* in stool samples of a larger cohort of volunteers/patients (>100 persons). Respective aptamer libraries and selected individual aptamers exist also for other prominent next‐generation probiotics like *A. muciniphila*,^[^ ^21^ ^]^ *R. intestinalis*,^[^ ^8^ ^]^ and *P. distasonis*,^[^ ^25^ ^]^ which suggest to implement respective gFET sensors also for quantification of these important microbiome members. Individual sensors or multiplexed variants of this detection technology may develop into an easy way to fast but reliable quantification of subsets of human gut microbiome bacteria in general, opening new avenues toward a supportive (bedside/home) monitoring of successful probiotic supplementation or future probiotic therapies of important diseases including neurodegenerative disorders with known dependency from dysbiosis of the gut microbiome composition.

## 3 Conclusion

Biofunctionalized gFETs have repeatedly been claimed to be a technology, which may serve as a precise sensing platform for a variety of biomedical and biotechnological applications. Simultaneously aptamers have shown their potential as valuable binding molecules to functionalize sensor surfaces. Here we show that bioinformatical analyses can easily deliver individual aptamers isolated from polyclonal SELEX aptamer libraries for which an applicability has already been demonstrated. These selected and chemically synthesized individual aptamers were biochemically characterized including methods like fluorescence labeling and subsequent binding assays to the *R. intestinalis* target cells also in the presence of defined mixtures of control gut bacteria. High affinities were determined with K_d_ values in the low nanomolar range, which qualifies them as suitable for specific high‐affinity binding of their target bacterium. These aptamers were also suitable for biofunctionalization of gFET sensors, allowing the specific quantification and hence discrimination of the desired target cells from the bacterial “contaminating” controls. The lower detection limit was found to be 10 cells mL^−1^ and specific measurements were possible in mixtures of *R. intestinalis* with these control bacteria. In the background of human stool samples alterations in the abundance of *R. intestinalis* in the range of 1% could be easily be retraced. This is sensitive enough to realistically promise the construction of a sensor device for the quantitative supportive monitoring of probiotic supplementation strategies in biomedicine.

## 4 Experimental Section

### Illumina® Sequencing of Aptamer Libraries and Structural Analysis of Representative Single Aptamers

The aptamer libraries Ri 4 and Ri 7_2 were sent for NGS Illumina amplicon sequencing to Eurofins Genomics (Konstanz, Germany). Therefore, the forward primer: 5´‐ACGATGATACTCGGACTGTAGGGAAGAGAAGGACATATGAT‐3′ and reverse primer: 5´‐TCTCGTGTTCAAGCGACTCAAGTGGTCATGTACTAGTCAA‐3′ were used for the first round of PCR amplification to introduce universal primer binding sites. The amplification reaction was carried out using Herculase II Fusion DNA Polymerases (Agilent Technologies, Inc., Santa Clara, California, USA) in a three‐step thermal cycle: initial denaturation at 95 °C for 3 min, followed by 9 cycles of denaturation at 95 °C for 30 s, annealing at 56 °C for 30 s, extension at 72 °C for 10 s, and a final extension at 72 °C for 2 min. Subsequently, the purified PCR products were used as templates for the second round of PCR to introduce index sequences for parallel sequencing of aptamer libraries. For the Ri 4 and Ri 7_2 libraries, primers 5′‐TCAGTCGTATATCACGACGATGATACTCGGACTG‐3′ and 5′‐GCTATGTACTCGTGATTCTCGTAGTTCAAGCGAC‐3′, as well as primers 5′‐TCAGTCGTATCGATGTACGATGATACTCGGACTG‐3′ and 5′‐GCTATGTACTACATCGTCTCGTAGTTCAAGCGAC‐3′, were used for differentiation. Subsequently, FastQC was used for quality control of NGS data,^[^ ^44^ ^]^ sequences were sorted, adapter sequences were trimmed using the FASTX toolkit, and nucleotide distribution analysis was performed. Finally, the secondary structure of the sequences was predicted using the FASTAptamer toolbox and the Mfold server.^[^ ^24^ ^]^

### Cultivation of Bacterial Species and Traditional Quantification by Cell Counting and Measurements of Optical Densities—Growth of Bacteria

The bacterial strains *R. intestinalis* (DSM‐14610), *P. distasonis* (DSM‐29491), *A. muciniphila* mucT (DSM‐22959), *A. stercoricanis* (DSM‐13633), *B. producta* (DSM‐29491) and *R. microfusus* (DSM‐15922) were cultivated in Schaedler Bouillon Medium (Carl Roth GmbH + Co. KG, Karlsruhe, Germany) at 37 °C under anaerobic conditions (90% N_2_ + 10% H_2_) in a Whitley A25 anaerobic work station (Meintrup DWS Laborgeräte GmbH, Herzlake, Germany).

### Cultivation of Bacterial Species and Traditional Quantification by Cell Counting and Measurements of Optical Densities—Determination of Cell Densities

The optical density (O.D._600_) was measured spectrophotometrically at a wavelength of λ = 600 nm in a UV‐1600 PC spectrophotometer (VWR International, LLC., Leuven, Netherlands).

### Cultivation of Bacterial Species and Traditional Quantification by Cell Counting and Measurements of Optical Densities—Determination of Cell Numbers

Cell numbers in bacterial cultures and flow‐throughs of gFET sensor measurements were determined by preparing serial dilutions and counting in a Neubauer Chamber in a Leica DMi8 coded (Leica Microsystems CMS GmbH, Wetzlar, Germany).

### Fluorescence‐Based Biochemical Characterization of Single Aptamers

Similar to previously described methods,^[^ ^8^ ^]^ the specificity, sensitivity, and affinity were analyzed using fluorescently‐labeled individual aptamers after chemical synthesis (biomers, Ulm, Germany). Also, quantitative tracking ability of *R. intestinalis* in mixed bacterial communities was performed for the selected individual aptamers.

### gfet Sensor Chip Quality Control

The gFET sensors ([ED‐IDE1‐Au], Micrux Technologies, Gijon, Spain) were coated with reduced graphene prepared accordingly to the previously established protocol,^[^ ^26^ ^]^ and the coating was verified afterward for each production batch.

### gfet Sensor Chip Quality Control—Synthesis of Bulk RGO

Graphene oxide water dispersion (200 µL) was centrifuged for 60 s at 10 000 rpm and the supernatant discarded. Precipitated GO was dried over night at 60 °C *in vacuo*. 50 mg of dried powder was weighed into a reaction tube, which then was placed in a glass container. After the addition of 100 µL hydrazine hydrate (80% in water) the glass container was tightly closed and incubated at 60 °C over night. Dried powders were analyzed via Raman spectroscopy.

### gfet Sensor Chip Quality Control—Raman Spectroscopy

The Raman‐measurements were performed on a confocal Raman microscope (WITec alpha300 R) utilizing a YAG‐laser (WITec, λ = 532 nm, 0.2 mW laser power) and a VIS spectrometer (WITec UHTS 300) with a spectral resolution of ± 1 rel. cm^−1^. The imaging of bulk powder of rGO, GO and sensor coated rGO was taken with a 50x and 100x ZEISS objective with a lateral resolution ≈2 px µm^−1^. The datasets were analyzed using the software Project 5.0 from WITec. Background correction was performed for each recorded spectrum using a shape filter (size: 400 pixels, circular shape), and the very intense peaks caused by cosmic radiation were removed. For all samples, measurements were performed at three different locations throughout a sample and are shown as a median.

### gfet‐Based Quantitative Measurements

For functionalization, 500 µm PyPEG (sealing agent polyethylene glycol with PBSE connector pre‐fix) and 50 µm 1‐pyridine carboxylic acid (PCA) were dissolved in DMSO. The gFETs were immersed in the mixture and reacted at room temperature in the dark for 24 h. Subsequently, the gFETs were washed twice with 1 mL of isopropanol and carefully dried under nitrogen. To monitor the bio‐recognition, the transfer characteristic of the gFET (I_D_V_G_) was recorded using a Keysight U2722A modular source/measure unit (Keysight Technologies, USA) and a custom developed LabView‐based software (National Instruments, USA). A specific range of gate voltages (−0.4 to +0.4 V) at a scan rate of 20 mV s^−1^ with V_DS_ = 50 mV was used as system parameters and the I_D_V_G_ curves were recorded. First, the chip surface was rinsed with distilled water at a flow rate of 0.2 mL mi^−^n^−1^ for 10 min, followed by rinsing with 0.01× DPBS at a flow rate of 0.5 mL mi^−^n^−1^ for 2 min. Afterward, the variation in surface current with respect to voltage was measured. Then, for aptamer‐coupling 1 mL of fresh 0.01× DPBS solution containing EDC (15 mm) and NHS (15 mm) was prepared and to the chips were rinsed at a flow rate of 0.2 mL min^−1^ for 30 min to fully activate the carboxyl groups on the chip surface. Then, the system was rinsed with 0.01× DPBS at a flow rate of 0.5 mL mi^−^n^−1^ for 1 min, followed by reducing the flow rate to 0.2 mL mi^−^n^−1^ and continuing to rinse for 10 min to completely remove any residual EDC and NHS. Then, 1 mL of 1× DPBS containing 100 pmol of activated amino‐labeled single aptamer was circulated at a flow rate to 0.2 mL mi^−^n^−1^ for 1 h, allowing the activated aptamer to fully attach to the chip surface, thereby completing chip functionalization. Again, the variation in surface current with respect to voltage was monitored to verify successful aptamer coupling. Finally, the target bacteria at desired cell numbers in 1 mL of 1× DPBS were send to the chip surface in the same for 15 min. Subsequently, the system was rinsed with 0.01× DPBS at a flow rate of 0.2 mL mi^−^n^−1^ for 5 min to remove unbound bacteria, and then binding was measured by recording the I_D_V_G_ curves as described above.

### Analysis of R. Intestinalis Abundance in Fecal Samples

Stool samples were collected from a healthy, lean volunteer who was recruited from Ulm University and provided written informed consent. The study was approved by the local ethics committee of Ulm University (reference number 30/20). In addition, the design and conduct of the study followed the regulations for the use of human research participants and strictly followed the standards set out in the Declaration of Helsinki. Stool samples were weighed, added to 1 mL of extraction buffer (1 × DPBS) and vortexed for 1 min until no stool particles were visible. Then, the crude fecal extract was filtered, centrifuged at 7500 × g for 1 min, and washed with 1 mL of 1× DPBS. Subsequently, the fecal bacteria were adjusted to an O.D._600_ = 1 and subjected to gGO‐FET biosensing with calculated increasing cell numbers of *R. intestinalis* (0 – 40 bacterial cells) in the background of 1000 fecal bacterial cells as calibration measurements, in which the specific increases correlated linearly with the shift increases for the aptamer sensors. The *R. intestinalis* content in stool samples was then analyzed using the individually derived linear curve.

### Analysis of R. Intestinalis Abundance in Fecal Samples—Next‐Generation Sequencing of Stool Sample Bacteria

The fecal samples were collected using INTEST.pro (Biomes Laboratory, Wildau, Germany) and then measured and analyzed by BIOMES laboratory (Wildau, Germany), using 16S rRNA/DNA NGS for fecal bacterial abundance, according to Lilja et al., 2021.^[^ ^45^ ^]^

### Statistical Analyses

All experiments were conducted as triplicate and statistical significance was determined using an unpaired Student's *t*‐test. *p* values < 0.05 were considered significant. ^*^ *p* denotes < 0.05, ^**^ denotes *p *< 0.01, ^***^ denotes *p *< 0.001 and ^****^ denotes *p *< 0.0001.

### Ethics Approval Statement

Fecal samples were obtained from a healthy, lean volunteer recruited from Ulm University, who provided written informed consent. The study was approved by the local ethics committee of Ulm University (reference number 30/20). Furthermore, the design and implementation of the study adhered to regulations of the German law for the use of human research participants and strictly followed the standards established by the Helsinki Declaration.

## Conflict of Interest

The authors declare no conflict of interest.

## Supporting information



Supporting Information

Supporting Table

## Data Availability

The data that support the findings of this study are available from the corresponding author upon reasonable request.
