# Supplementary material for: Specific gFET‐Based Aptasensors for Monitoring of Microbiome Quality: Quantification of the Enteric Health‐Relevant Bacterium Roseburia Intestinalis
Source: Adv Healthc Mater. 2024 Dec 11;14(4):2403827. doi: 10.1002/adhm.202403827 (PMC11804837; doi:10.1002/adhm.202403827)
Supplement: Supplementary file 1 — Supporting Information [file ADHM-14-0-s002.docx]

**Specific gFET-Based Aptasensors for Monitoring of Microbiome Quality: Quantification of the Enteric Health-Relevant Bacterium *Roseburia intestinalis***

*Yiting Zhang,^a,†^ Hu Xing,^a,†^ Runliu Li,^a^ Jakob Andersson,^b^ Anil Bozdogan,^c^ Robert Strassl,^c^ Bastian Draphoen,^d^ Mika Lindén,^d^ Marius Henkel,^e^ Uwe Knippschild,^f^ Roger Hasler,^g^ Christoph Kleber,^g^ Wolfgang Knoll,^g^ Ann-Kathrin Kissmann^a,^* and Frank Rosenau^a,^**

^a^ Institute of Pharmaceutical Biotechnology, Ulm University, Albert-Einstein-Allee 11, 89081 Ulm, Germany

^b^ AIT Austrian Institute of Technology GmbH, Giefinggasse 4, 1210 Vienna, Austria

^c^ Division of Clinical Virology, Medical University of Vienna - Spitalgasse 23, 1090 Vienna, Austria

^d^ Institute of Inorganic Chemistry II, Ulm University, Albert-Einstein-Allee 11, 89081 Ulm, Germany

^e^ Cellular Agriculture, TUM School of Life Sciences, Technical University of Munich, Gregor-Mendel-Str. 4, 85354 Freising, Germany

^f^ Department of General and Visceral Surgery, Surgery Center, Ulm University, Albert-Einstein-Allee 23, 89081 Ulm, Germany

^g^ Danube Private University, Steiner Landstraße 124, 3500 Krems an der Donau, Austria

* Correspondence: ann-kathrin.kissmann@uni-ulm.de (A.-K.K.); frank.rosenau@uni-ulm.de (F.R.)

† These authors contributed equally to this work.

Supplementary Information

**
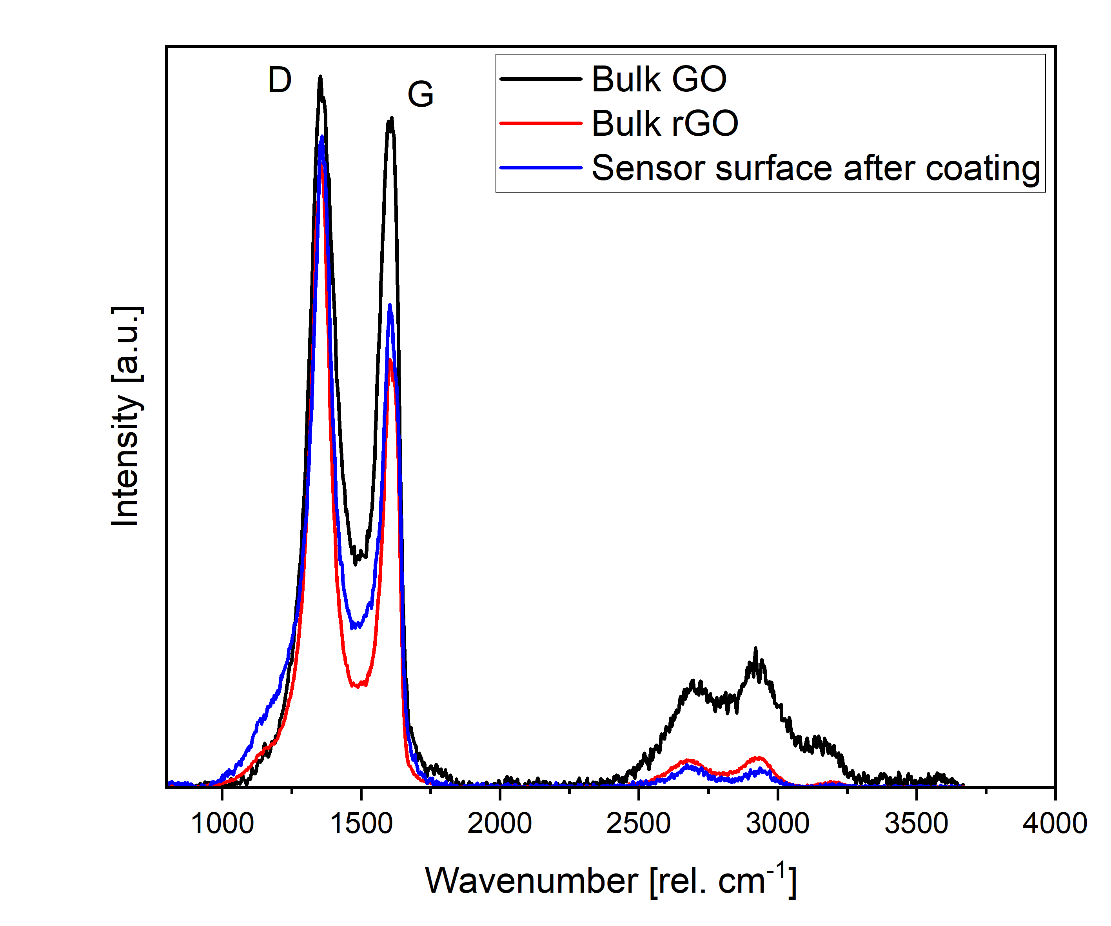
**

**Figure S1.** Raman spectroscopy of bulk GO (black), bulk rGO (red) and a GO coated and reduced sensor surface (blue) with a 532 nm laser with a laser power of 200 µW. Data is shown as the mean of three different Raman spectra of three different locations throughout the samples. Increase of I_D_/I_G_-ratio from bulk GO (I_D_/I_G_=1.06) to bulk rGO (I_D_/I_G_=1.46) and sensor surface after coating (I_D_/I_G_=1.34) proves reduction of GO with hydrazine in accordance with literature.^[1]^

**
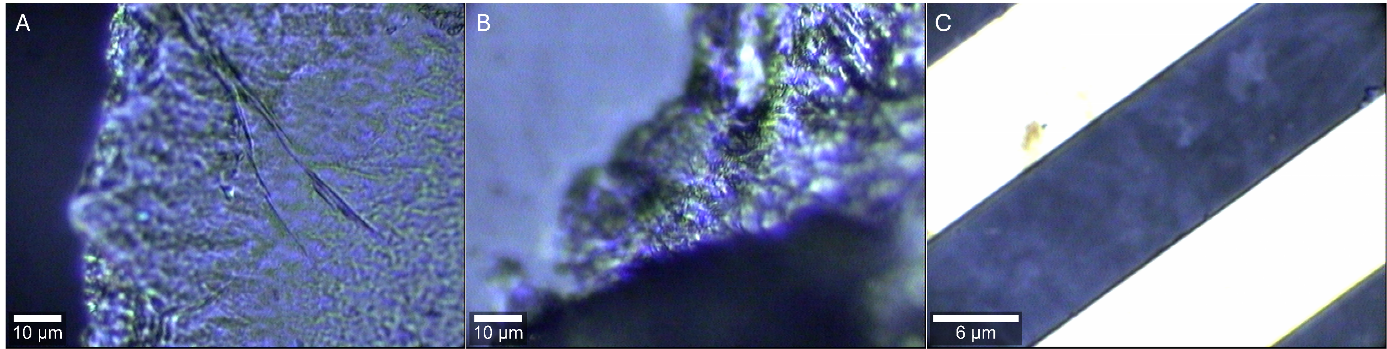
**

**Figure S2.** Microscopy images of bulk GO (A), bulk rGO (B ) at a 50x magnification and the sensor surface (C) at a 100x magnification.

**References**

[1] I. K. Moon, J. Lee, R. S. Ruoff, H. Lee, *Nature Communications* **2010**, *1*, 73.
